# Addendum: Clinical trial registration number for interventional health studies

**DOI:** 10.4069/kjwhn.2022.12.21

**Published:** 2022-12-29

This addendum was issued to note clinical trial registration numbers for the following publications. In line with strengthening journal guidelines on adhering to the ICMJE guidelines on specifying clinical trial registration for interventional studies, the following manuscripts declare their clinical trial registration numbers. Prospective registration was not mandated by the funding agencies at start of the study and thus, retrospective registration was done by the author(s).

Kim SH. Development and effects of a webtoon education program on preventive self-manage­ment related to premature labor for women of childbearing age: a randomized controlled trial. Kore­an J Women Health Nurs. 2022;28(3):250-263. https://doi.org/10.4069/kjwhn.2022.09.22

→ KCT0008015

Kim SM, Ahn S. Development and application of a self-transcendence enhancement program for the well-being of elderly women living alone in Korea. Korean J Women Health Nurs. 2021;27(2):128-140. https://doi.org/10.4069/kjwhn.2021.06.07

→ KCT0008010

The clinical trial registration number has also been updated to the manuscript PDF and online access.

*Korean Journal of Women Health Nursing* strongly recommends prospective registration of interventional health studies.

